# Navigating the Twist: An Atypical Presentation of Malrotation

**DOI:** 10.1055/a-2803-3478

**Published:** 2026-03-05

**Authors:** Shivangi Ganeshan, Kimberley R. Zakka, Arash R. Zandieh, Manuel B. Torres, Lewis P. Rubin

**Affiliations:** 1 Department of Pediatrics, MedStar Georgetown University Hospital, Washington, District of Columbia, United States; 2 Department of Radiology, MedStar Georgetown University Hospital, Washington, District of Columbia, United States; 3 Georgetown University School of Medicine, Washington, District of Columbia, United States; 4 Department of Pediatric Surgery, MedStar Georgetown University Hospital, Washington, District of Columbia, United States; 5 Department of General and Thoracic surgery, Children's National Hospital, Washington, District of Columbia, United States; 6 Division of Neonatal-Perinatal Medicine, MedStar Georgetown University Hospital, Washington, District of Columbia, United States

**Keywords:** neonate, GI bleeding, malrotation, atypical presentation

## Abstract

Intestinal malrotation is a congenital anomaly resulting from abnormal midgut rotation and fixation and occurs in approximately 1 in 500 live births. Malrotation results in a narrow mesenteric root, predisposing to midgut volvulus and potentially life-threatening bowel ischemia. Symptoms develop in about 1 in 6,000 individuals, over 75% of cases presenting in the early neonatal period. Bilious vomiting and abdominal distension are common signs of presentation.

We report a case of a healthy term male neonate who was breastfeeding with formula supplementation until day of life 3, when he passed two bloody stools. He was transferred to our neonatal intensive care unit for evaluation and management. Abdominal ultrasound demonstrated pathognomonic reversal of the relationship between the superior mesenteric artery and superior mesenteric vein and an upper gastrointestinal (GI) contrast study confirmed intestinal malrotation. He underwent an urgent exploratory laparotomy and corrective Ladd procedure. There was no intraoperative evidence of volvulus or bowel ischemia. He had an uncomplicated recovery and was discharged several days later.

GI bleeding is a rare initial presentation of malrotation, particularly in the absence of bilious emesis. This case emphasizes the importance of considering malrotation in neonates with hematochezia to enable early diagnosis and prevent life-threatening complications.

## Introduction

Intestinal malrotation is a congenital anomaly resulting from an abnormal developmental sequence of bowel rotation and fixation during fetal development. When complicated by volvulus, the resulting compromise in mesenteric blood flow rapidly can lead to bowel ischemia, necrosis, and, potentially, perforation—conditions that may be fatal if not promptly diagnosed and treated. Bilious emesis is the most common presenting symptom, occurring in an estimated 70 to 90% of cases of symptomatic neonatal malrotation with midgut volvulus. 1 2 We present the case of a newborn with malrotation who was admitted with hematochezia as the sole presenting symptom. This case highlights the importance of recognizing atypical presentations of this potentially life-threatening condition.

## Case Description

This male infant was born at 38 weeks and 3 days of gestation to a 40-year-old primigravid mother by cesarean section due to a nonreassuring fetal heart tracing. The pregnancy was conceived through in vitro fertilization and maternal history was notable for advanced maternal age, chronic hypertension, obesity, and prediabetes. All prenatal laboratory tests were normal. The mother received perioperative antibiotic prophylaxis with cefazolin and azithromycin.

The infant had Apgar scores of 8 and 9 at 1 and 5 minutes of life, respectively. Immediate postnatal resuscitation included continuous positive airway pressure for 5 minutes. He initially breastfed well but began formula supplementation on day of life (DOL) 3 due to a 9.5% weight loss. There were no reports of emesis. However, on DOL 3, he had two episodes of bloody stools. An initial abdominal X-ray revealed a nonobstructive bowel gas pattern, and he was transferred to our facility for further evaluation and management.

**Fig. 1:**
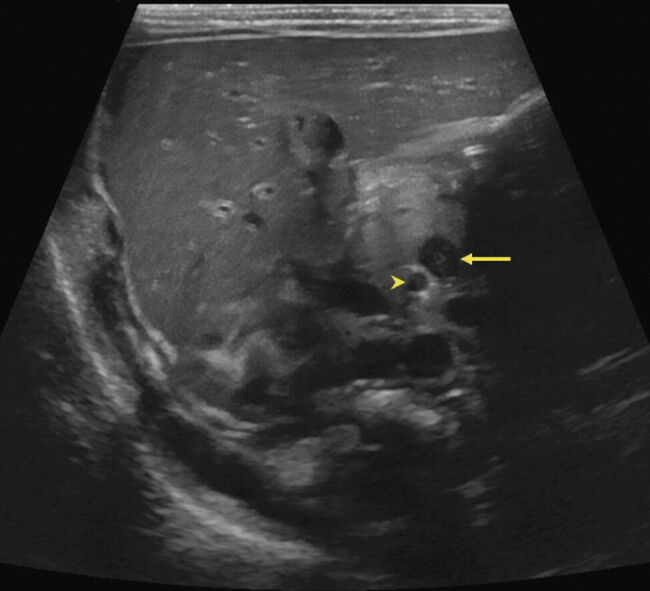
Abdominal ultrasound. Focused sonographic imaging of the mid abdomen shows reversal of the normal SMA to SMV relationship, indicating intestinal malrotation. The SMV (arrow) is located to the left of the SMA (arrowhead), which is a reversal of the normal SMA/SMV relationship. Real-time sweep of the mesenteric axis showed swirling of the mesentery around the SMA (not shown). SMA, superior mesenteric artery; SMV, superior mesenteric vein.

**Fig. 2:**
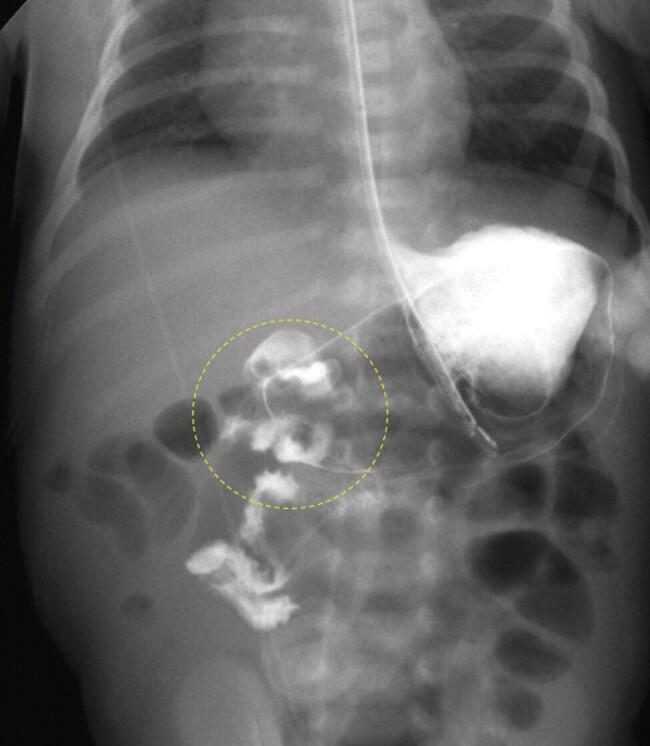
Upper GI series. The proximal small bowel resides in the right hemiabdomen and does not cross the midline, consistent with malrotation. The ligament of Treitz could not be documented. There was suspicious swirling of the duodenum (circle), raising concern for midgut volvulus. The stomach was distended, and several episodes of gastroesophageal reflux were observed (not shown). GI, gastrointestinal; SMA, superior mesenteric artery; SMV, superior mesenteric vein.

On arrival, he was well-appearing with a soft but distended abdomen. Bowel sounds were hypoactive, and there was no overlying discoloration. Laboratory evaluation was notable for mild hypernatremia (Sodium 149) and hyperchloremia (Chloride 118). A repeat abdominal X-ray showed dilated, gas-distended loops of bowel in the right upper quadrant, raising concern for a developing bowel obstruction. A targeted abdominal ultrasound (US) ( Fig. 1 ) showed reversal of the normal relationship between the superior mesenteric artery (SMA) and vein (SMV). An upper gastrointestinal (UGI) contrast study demonstrated an abnormally positioned duodenojejunal junction, confirming intestinal malrotation ( Fig. 2 ).

The patient underwent urgent exploratory laparotomy and a corrective Ladd procedure. There was no intraoperative evidence of volvulus or bowel ischemia, but a small mesenteric hematoma was present. Genetic testing was considered, but not pursued, given the isolated anomaly and the absence of other associated features of a possible underlying genetic disorder. He had an uncomplicated postoperative course and was discharged home a few days later with close surgical follow-up.

## Discussion

During normal embryologic development, the midgut undergoes a 270-degree counterclockwise rotation around the SMA between the 6 ^th^ and 10 ^th^ weeks of gestation. This rotation ensures proper positioning and fixation of the small and large intestines within the abdominal cavity. In malrotation, this process is disrupted, resulting in incomplete or absent counterclockwise rotation of the midgut, leading to an abnormally positioned duodenojejunal junction and cecum and a shortened and narrow mesenteric root. This narrow mesenteric vascular bundle predisposes to intermittent midgut torsion around its blood supply and potentially life-threatening volvulus. Malrotation occurs in approximately 1 in every 500 live births, with associated volvulus occurring in an estimated 1 in every 2,500 live births. 3 4 5 6 Intestinal malrotation may be associated with other congenital anomalies, including small bowel atresia, anorectal malformations, duodenal webs, cardiac defects, and trisomy 21. 7

A maxim of neonatal care is bilious vomiting is a diagnostic and potentially surgical emergency, since it may be the presenting sign of midgut volvulus due to intestinal malrotation. Bilious vomiting variously indicates volvulation around the narrow mesenteric pedicle, causing twisting of the SMA and SMV, compression of the duodenum by peritoneal (Ladd) bands, or an associated duodenal web or atresia. Nevertheless, in several prospective audits of cases of bilious vomiting in neonates, mostly surveying term neonates, 8 9 10 only 9 to 16% of episodes were caused by intestinal malrotation.

Malrotation typically presents in term neonates within the first few days of life, the diagnosis most often prompted by bilious emesis. Acute intestinal obstruction secondary to volvulus may rapidly progress to venous congestion, ischemia, and hemorrhagic necrosis of the affected bowel. Frankly, bloody stools can be a late sign suggestive of advanced vascular compromise. 11 12

Malrotation with midgut volvulus constitutes a surgical emergency; 80 to 100% of cases present with bilious emesis, 1 2 4 9 13 14 15 16 and many demonstrate signs of shock once ischemia 14 is present. In contrast, in older children, the clinical picture may be more variable and include intermittent abdominal pain, episodic (and often nonbilious) vomiting, or poor weight gain—symptoms that sometimes are misattributed to gastroesophageal reflux. 1 7

GI bleeding in neonates is relatively rare and encompasses a wide range of pathologies. 17 Common causes include anal fissures, swallowed maternal blood, ulcers/erosions, and milk protein allergy or enterocolitis. 18 19 Less commonly, GI bleeding is caused by vascular malformations, coagulopathies, GI duplications, Hirschsprung's disease, or malrotation. The rare co-occurrence of intestinal malrotation and acute intussusception, known as Waugh's syndrome, also may include rectal bleeding. 20

In older children, occult GI bleeding has been reported as a less common presentation of partial, chronic, or intermittent volvulus. 16 In contrast, rectal bleeding as the presenting sign of intestinal malrotation in a neonate has rarely been reported. 21 Distinguishing between upper and lower GI sources is essential, as is promptly recognizing life-threatening conditions. In a 25-year review by Andrassy and Mahour, 74 cases of malrotation were identified, 30 involving midgut volvulus. All presented with bilious emesis and signs of high intestinal obstruction, and only eight had associated GI bleeding. 13

The patient in our case had an atypical presentation of malrotation, with two episodes of hematochezia and no reported vomiting. He showed no signs of feeding intolerance or systemic instability. Abdominal US and UGI contrast studies confirmed intestinal malrotation. Exploratory laparotomy revealed malrotation without evidence of volvulus, bowel ischemia, or necrosis. However, a small mesenteric hematoma was suspected to be related to intermittent volvulus and potentially accounted for the observed bleeding.

Traditionally, an UGI series has been the gold standard for evaluating suspected malrotation in neonates presenting with bilious emesis, offering over 90% diagnostic accuracy. 3 22 However, a growing body of evidence indicates that US has greater diagnostic accuracy than UGI in this context, 23 24 25 particularly the identification of abnormal SMA/SMV relationships and the “whirlpool sign.” 26 A common diagnostic approach for the diagnosis of malrotation and volvulus is the use of UGI with US as an adjunct. 10 27

Timely diagnosis of intestinal malrotation is critical, as delays can lead to catastrophic complications, 28 including bowel necrosis, extensive resections resulting in short bowel syndrome, dependence on long-term parenteral nutrition, need for intestinal transplantation, or death from sepsis.

## Conclusion

Recognizing atypical presentations of common GI emergencies in the neonatal period is crucial. Maintaining a high index of suspicion enables timely diagnosis and intervention, which is essential to prevent potentially devastating complications.
